# A case of primary conjunctival follicular lymphoma presenting as nasolacrimal obstruction

**DOI:** 10.1093/jscr/rjad616

**Published:** 2023-11-12

**Authors:** Amaar Amir, Baraa Amir, Salwa Sheikh

**Affiliations:** College of Medicine, Imam Abdulrahman Bin Faisal University, Dammam 34212, Saudi Arabia; College of Medicine, Imam Abdulrahman Bin Faisal University, Dammam 34212, Saudi Arabia; Pathology Services, John Hopkins Aramco Healthcare, Dhahran 34455, Saudi Arabia

**Keywords:** ocular adnexal lymphoma, follicular lymphoma, conjunctiva, nasolacrimal obstruction

## Abstract

We herein report a 76-year-old male who presented to the ophthalmology clinic after complaining from excessive lacrimation for ~6 months prior to presentation. He reports previously undergoing a nasolacrimal recanalization procedure in attempts to resolve his symptoms, but reported no improvement. On examination, a mass on the nasal conjunctiva around the medial canthus of the right eye was noted and subsequently excised. The findings support the diagnosis of low-grade follicular lymphoma. The patient was referred to radiation oncology for radiation therapy and received a total dose of 2400 cGy. Most ocular adnexal lymphomas are B-cell in origin, with follicular lymphomas being one of the rarest forms of such lymphomas. The most common translocation reported in over 85% of follicular lymphomas of the ocular adnexa is t(14; 18) (q32; q21). Traditional treatment options typically include a mix of chemical, surgical, and radio-oncological interventions.

## Introduction

Ocular adnexal lymphomas (OALs), which involve the tissue surrounding the eye and optic nerve, is a rarely encountered entity, with ocular adnexal follicular lymphomas being one of the least encountered. We herein report an elderly gentleman who presented to the ophthalmology clinic after complaining from excessive lacrimation for ~6 months prior to presentation. He reports previously undergoing a nasolacrimal recanalization procedure in attempts to resolve his symptoms, but reported no improvement. Examination revealed a mass on the nasal conjunctiva around the medial canthus of the right eye. The mass was subsequently excised and histopathological examination supported the diagnosis of low-grade follicular lymphoma. The patient was referred to radiation oncology for radiation therapy and received a total dose of 2400 cGy. On a 6 month follow-up, the patient was found to be in remission, though he does complain that the watery discharge has not yet completely returned to baseline.

## Case presentation

A 76-year-old male known case of hypertension and diabetes mellitus type 2 presented to the ophthalmology clinic after complaining from excessive lacrimation for ~6 months prior to presentation. He reports previously undergoing a nasolacrimal recanalization procedure in attempts to resolve his symptoms, but reported no improvement. The patient denied any history of fever, chills, night sweats, and significant weight loss. Similarly, the patient denied any new onset vision loss, ocular pain and redness, and photophobia. The patient’s remaining medical and surgical history is otherwise not significant. On examination, a mass on the nasal conjunctiva around the medial canthus of the right eye was noted and subsequently excised.

**Figure 1:**
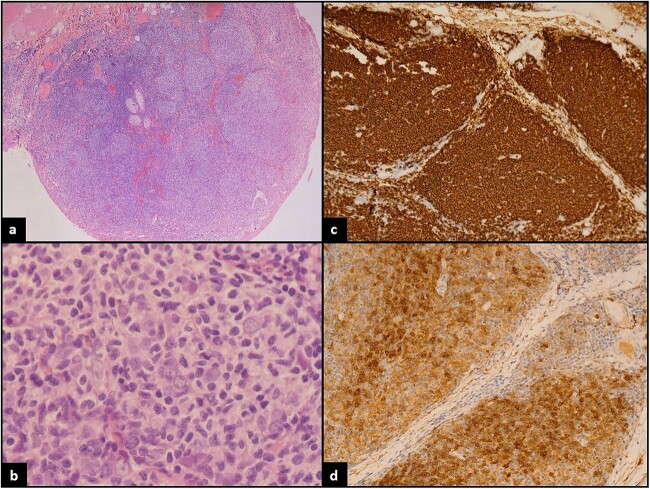
(**a**) Low power: conjunctival mucosa with underlying neoplastic lymphoid proliferation showing nodular architecture. (**b**) High power: atypical small to medium in size lymphocytes with cleaved nuclei. (**c**) CD20 strongly positive by most of the lymphoma cells confirming B lineage. (**d**) CD10 positive in the lymphoma cells.

Histopathological examination revealed effacement of the normal architecture and replacement by closely packed variable sized and shaped non-polarized follicles that are evenly distributed throughout the specimen. The mantle zone is generally attenuated. Most of the follicles revealed monomorphic appearance and are predominantly composed of small lymphoid cells with both cleaved and noncleaved centrocytes. Most of the nuclei are characteristically twisted, indented, elongated, and angulated with inconspicuous nucleoli. Centroblasts constitute <16 cells per 400× field on average within the nodules. Immunoperoxidase studies were performed and revealed that the atypical lymphocytes mark as CD20-positive B-lymphocytes that coexpress CD10, BCL6, and LMO2, and aberrantly coexpress BCL2 (Fig. 1). The nodules are centered on CD21-positive follicular dendritic cell meshworks. CD3 highlights reactive background T-lymphocytes. The findings support the diagnosis of low-grade follicular lymphoma. The patient underwent a Positron Emission Tomography and Computed Tomography (PET CT) 1 week after the excisional biopsy and showed mild tracer avidity around the area of excision which may represent post procedural changes or residual disease. The patient was referred to radiation oncology for radiation therapy where a total dose of 2400 cGy was successfully delivered in 12 fractions over 2 weeks. On a 6 month follow-up, the patient was found to be in remission, though he does complain that the watery discharge has not yet completely returned to baseline.

## Discussion

OALs are rare tumors involving the conjunctiva, lacrimal drainage system, eyelids, and ocular muscles [1]. Of the varying structures comprising the ocular adnexa, the orbit is most commonly involved with a reported rate of 46%–74%. The conjunctiva and eyelid are reported to be the least frequently involved at 20%–33% and 5%–20%, respectively [2]. Most OALs of B-cell origin tend to exhibit unilateral involvement with the exception of MCL. OALs may present as primary or secondary lesions if accompanied with an identical form of lymphoma present systemically. However, one-third of patients presenting with primary OALs are reported to develop systemic lymphoma within a decade. Most OALs are Non-Hodgkin lymphoma (NHL), with an overwhelming majority possessing B-cell origin. The most common subtype is extranodal marginal zone B-cell lymphoma (EMZL), followed by the less frequently reported subtypes of diffuse large B-cell lymphoma (DLBCL), follicular lymphoma (FL), and mantle cell lymphoma (MCL). While reports do vary among the literature, one international multicenter review article investigated 2211 cases of orbital lymphoma between 1994 and 2017. Of the 2211 cases, 2139 cases (97%) were B-cell lymphomas with EMZL being the most common subtype reported (59%), followed by DLBCL (23%), FL (9%), and MCL (5%). Of the remaining cases, 72 were T-cell in origin and 46 cases were mixed T-cell and NK-cell in origin [3].

Generally, it has been noted that OALs primarily affect older individuals. This is especially true in regards to OALs of B-cell origin. In contrast, OALs from T-cell or NK-cell origin tend to show a more varied age distribution. Gender distribution is often seldom reported, though a female predominance has been reported in cases of EZML (53%) and FL (75%). On the other hand, MCL displays a striking male predominance (80%). Similarly, the duration of patient symptoms prior to ophthalmology consultation varies significantly according to the subtype of OAL involved; however, prolonged median symptoms duration for all OALs is generally reported. FL was reported to have a median duration of symptoms of 24 months. This was considerably longer than EMZL, DLBCL, and the T-cell lymphomas which typically sought out consultation within weeks or a few months. One of the primary reasons for delayed consultation is misdiagnosing OALs which can delay the correct diagnostic process. Typical presenting symptoms can range considerably and include restricted eye mobility, pain, change in visual acuity, diplopia, erythema, edema, swelling or mass, proptosis, excessive lacrimation, and B symptoms. Staging the disease using the Ann Arbor classification is of utmost importance in selecting the appropriate management [3, 4].

FLs of the ocular adnexal demonstrate a complex and multifactorial pathogenetic makeup. The most common translocation reported in over 85% of FL of the ocular adnexa is t(14; 18) (q32; q21). This aberration results in an inappropriately functioning BCL2 oncogene and overexpression of BCL2 protein, which ultimately increases the survivability of affected malignant cells. Additional secondary genetic aberrations are typically necessary in the development of FL which may include losses of 1p, 6q, 10q, 13q, and gains of 1q, 2p, 8q, 12q, and 18q. In addition, ~30% of FL patients possess genomic alteration to the BCR/NF-kB signaling pathway. The aforementioned aberrations are reported to impact the process of transcription regulation as well as the signaling, differentiation, proliferation, and apoptosis of affected cells [5]. FL without the hallmark BCL2 translocation is a rare entity with a different clinical presentation and behavior typically demonstrating diffuse tissue involvement. Despite the critical importance of the hallmark t(14; 18) translocation in the pathogenesis of FL, the drug Venetoclax, a BCL2 inhibitor, has performed underwhelmingly according to several studies with an extremely high discontinuation rate reported [6]. Several other targeting therapies have performed better including Tazemetostat, an EZH2 inhibitor, with fewer reports of adverse effects and increased response rate. Other examples of targeting immunomodulatory therapies include monoclonal anti-CD47, CD20, and CD3 antibodies [5, 7].

Traditional treatment options typically include a mix of chemical, surgical, and radio-oncological interventions. A combined approach is superior to surgery-only management plans as the recurrence rate is generally lower. However, some studies reported that surgery alone may suffice in certain patients depending on the location and extent of the lymphoma. Radiotherapy is the gold standard for patients presenting with stage 1 OALs with patient specific doses typically ranging from 25 to 25 Gy [4]. Chemotherapy is especially important in cases of OALs with systemic involvement, with combination regimens including cyclophosphamide, adriamycin, vincristine, and prednisone, with or without rituximab (CHOP/R-CHOP) commonly used [8]. The prognosis of patients with FL of the ocular adnexa is generally favorable for low-grade lesions. Poor prognostic factors include primary involvement of regions other than the conjunctiva, advanced stage, older age at presentation, presence of B symptoms, and expression of CD5, CD43, p53, BCL6, and BCL10 [5].

## Data Availability

The data used to support the findings of this study are included within the article.
